# Estimated Burden of Serious Fungal Diseases in Serbia

**DOI:** 10.3390/jof4030076

**Published:** 2018-06-25

**Authors:** Valentina Arsić Arsenijević, David W. Denning

**Affiliations:** 1 National Reference Laboratory for Medical Mycology, Institute of Microbiology and Immunology, Faculty of Medicine, University of Belgrade, Dr Subotića Street 1, 11000 Belgrade, Serbia; 2 The National Aspergillosis Centre, University Hospital of South Manchester, The University of Manchester and Manchester Academic Health Science Centre, Manchester M13 9PL, UK; david.denning@manchester.ac.uk

**Keywords:** epidemiology, serious fungal infections or diseases, Serbia

## Abstract

For the first time, we aimed to estimate the burden of serious fungal infections or diseases (SFD) and highlight national epidemiological features in Serbia. Data on population and underlining conditions were extracted from the Statistical Office of the Republic of Serbia, World Bank, the Institute of Public Health of Serbia, the World Health Organization, National reference laboratory for medical mycology, the national registries of Serbian professional societies, and relevant publications. The population structure/inhabitants in 2016 (not including the autonomous region Kosovo & Metohija) was 7,058,322; with 6,041,743 adults (85.6%). The populations at risk (total cases per year) were: HIV infected 2441; acute myeloid leukemia 212; stem cell transplantation 151; solid organ transplants 59; chronic obstructive pulmonary disease 250,302; adult asthmatics 311,806; adult cystic fibrosis 65; pulmonary tuberculosis 898; lung cancer 7260; intensive care unit admissions 19,821; and renal support 520. Annual fungal disease cases estimated are: candidemia 518; invasive aspergillosis 619; *Candida* peritonitis 187; *Pneumocystis jirovecii* pneumonia 62; cryptococcosis 5; mucormycosis or fusariosis 23; severe asthma with fungal sensitization 10,393; allergic bronchopulmonary aspergillosis 9094; chronic pulmonary aspergillosis 448, recurrent *Candida* vaginitis 135,303; oral candidiasis 208,489; esophageal candidiasis 173, fungal keratitis 70; tinea capitis 300; and onychomycosis 342,721. We expect that 156,825 people suffer from serious SFD each year (2221/100,000), and 409 dies annually. Additionally, the prevalence of superficial infections exceeds 1,008,995 cases (14,295/100,000). The first *Rhinosporidium* outbreak in Europe was associated with Serbian Silver Lake. The plant pathogen *Fusarium* seems to be emerging in Serbian pediatric haematooncology settings. *Candida auris* and endemic mycoses have not been observed to date. These general estimates provide a primer for further efforts to study fungal epidemiology in Serbia.

## 1. Introduction

Fungi are of great medical importance in Serbia because they may cause serious fungal diseases (SFD), which can be either superficial (with chronic course and high morbidity), affecting quality of life (QoL) or invasive (difficult to diagnose and treat), significantly affecting life and with a high mortality rate. Generally, SFD usually occur in defined high-risk populations (HRP) such as low-birth-weight infants and severely immunocompromised hematological or transplant patients [1], although lung disease is a common antecedent also. Allergic bronchopulmonary aspergillosis (ABPA) complicates asthma and may lead to chronic pulmonary aspergillosis (CPA), yet the burdens of each have never been estimated in many countries [2]. However, the epidemiology of SFD is poorly documented in Serbia since no studies were done to determine the incidence or prevalence of SFD. Although sporadic data from individual centers about the incidence of *Candida* bloodstream infections (BSI) have been published [3], the national database of SFD is still under construction. The Global Action Fund for Fungal Infections (GAFFI, www.gaffi.org) and Leading International Fungal Education (LIFE, http://life-worldwide.org) have put a lot of effort into the estimation of national and global burdens of SFD. Many countries worldwide have used their methodology and published estimates of the SFD rates, including countries from the Mediterranean region [4,5,6] and Balkan countries [7,8].

Serbian mycology dates from almost hundred years back when one of the oldest specialized medical mycology laboratories in Europe was established within the Central Institute for Hygiene in Belgrade (1930) by Professor Sima Milochevitch. He did significant work on the education, research, surveys, diagnosis, treatments, and prevention of fungal diseases, and made a key discovery in medical mycology by proposing the laboratory criteria for dermatophyte classification. Since he did not survive the Second World War a huge gap in the systemic survey of fungal diseases then occurred.

In 2008, the National Reference Laboratory for Medical Mycology (NRL MM) and Serbian Society of Medical Mycology (SSMM, www.mikologija.org.rs) were established within Belgrade University. This development has made the field more dynamic and visible, improving mycology knowledge, identifying local problems, and overcoming major gaps in diagnostics. Over the last ten years clear progress in medical mycology is visible with the exchange of ideas occurring internationally and collaborations with ECMM (https://www.ecmm.info), ISHAM (https://www.isham.org), GAFFI, and LIFE [9]. 

Significant problems and gaps still need to be overcome, and in this study we aimed: (i) to estimate the burden of SFD in Serbia, using LIFE methodology, mostly by identifying rates in underlying populations at risk; (ii) to identify key specific local data of the importance for national estimation and underline some unusual features of Serbian fungal local pathology.

## 2. Materials and Methods

Relevant data regarding Serbia were downloaded from Wikipedia [10]. Statistical information about the Serbian population and structure was obtained from the Statistical Office of the Republic of Serbia and the estimation of SFD was mostly done based on national population data from 2016 [11]. The number of patients with HIV/AIDS was derived from the data of the Institute of Public Health of Serbia (IPHS) (Annual report about communicable diseases in 2016 in Republic Serbia), Joint United Nations Programme on HIV/AIDS (UNAIDS), and the World Health Organization (WHO). The WHO Tuberculosis (TB) surveillance and monitoring in Europe 2017 report and publications were used to obtain data on TB patients (ECDC, https://ecdc.europa.eu/en/home). The prevalence of hematological malignant (HM) diseases, as well as organ and tissue transplantation procedures, were partially derived from limited data from IPHS, and mostly from personal communications. The number of intensive care units (ICU) beds was collected from the database of the Serbian Association of Anesthesiologists and Intensives, and by personal communications. The number of patients with renal support was collected from the Serbian Society of Nephrology database, and by personal communications. The number of patients with asthma and chronic obstructive pulmonary disease (COPD) was partly obtained from IPHS (National population investigation in 2006) and by personal communications. Information about cystic fibrosis (CF) incidence was derived from the National Center for CF of Serbia. The data regarding population based surveys, documented fungal outbreaks, strain prevalence, strain distributions, and the number of high risk patients tested for fungal biomarkers were found in the database of Serbian NRL MM, and SSMM (2008–2018).

Where no national-level data were available, we reviewed published single- or case-series reports and single- or multi-center datasets for certain diseases. We performed an extensive review of the literature and extracted epidemiological surveys of the national SFD incidence rates, focusing on neighboring countries Hungary [7], and Romania [8] or on countries with a similar number of citizens Israel [5], and Greece [4]. Where no available data were found in any database, and in relevant published literatures, authors used local unpublished data and/or contacted national experts for certain diseases.

SFD estimates were generated according to previously proposed principles [12]. Generally, disease estimates were conservative as they assumed the lowest incidence rates reported in the literature and focused only on well-defined risk populations. For superficial, mucosal, and allergic, such as severe asthma with fungal sensitization (SAFS) and ABPA, we estimated the national burden and prevalence rates (number of persons living with fungal disease per 100,000 populations). For HIV associated oral candidiasis (OC) and esophageal candidiasis (EC) and life threatening fungal diseases, such as candidemia, invasive pulmonary aspergillosis (IPA) or rare/emerging fungi, the annual incidence rates were calculated.

## 3. Results

### 3.1. The Important Facts and Demographics of Serbia (Officially the Republic of Serbia)

**Figure 1:**
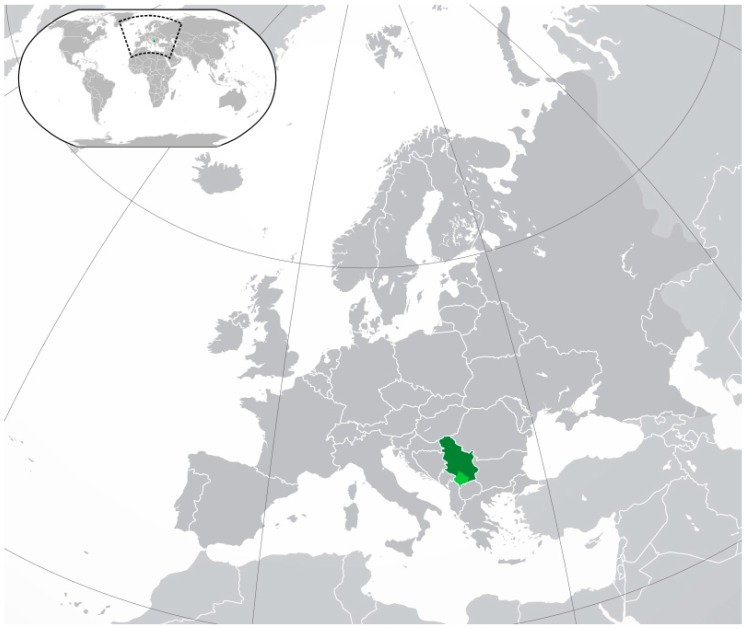
Location of Serbia (dark grey) in the Europe (grey) [10].

Serbia is located at the crossroads between Central and Southern Europe, in the heart of Balkan Peninsula and the south of the Pannonian Plain (Figure 1). The country covers a total of 88,361 km^2^, including the autonomous province Kosovo & Metohija (K&M), and bordered with Hungary, Romania, Bulgaria, the former Yugoslav Republic of Macedonia, Albania, Montenegro, Bosnia and Herzegovina, and Croatia. Agriculture is an important section of the Serbian economy, mostly prominent in Northern Serbia on the fertile Pannonian Plain. Serbia has many rivers and lakes and the river Danube passes through Serbia. The Gross Domestic Product per capita is 5852 US dollars, and Serbia is classed as an upper-middle income economy (2016). The current population of Serbia is 8,766,759, which is equivalent to 0.11% of the total world population. The median age is 40.2 years and the life expectancy is 75.1 years: male 72.6; female 77.7 (2016). Serbia is not a member of the European Union and its membership status is “candidate country”.

K&M declared their independence from Serbia in 2008, which Serbia rejects. However, Serbia does not control whole territory and in this study the data will be presented excluding K&M, based on a population of 7,056,322 citizens in 2016 (Table 1).

During the last hundred years, Serbia was destroyed by wars several times. The latest followed air NATO strikes during 1999 and some sites in K&M and the southern part of central Serbia were bombed with depleted uranium. After that 50 Italian soldiers died, having been in K&M at that time, and both their families and experts believe that exposure to depleted uranium weapons was responsible for their deaths. The use of depleted uranium munitions immediately preceded a dramatic jump in cancer incidence in central parts of Serbia over the last twenty years, but official data are difficult to obtain.

### 3.2. Serious Fungal Diseases (SFD) Affecting the Quality of Life (QoL)

The most common SFD affecting Serbian patients QoL are the ones localized on the skin, nail, hair, or cornea, namely: onychomycosis (OM), tinea capitis (TC), corporis, and fungal keratitis (FK). In addition, some diseases associated with mucosa such as recurrent vulvovaginal candidiasis (RVVC), oral-esophageal candidiasis (OC, EC), and some respiratory fungal conditions such as ABPA and SAFS may significantly affect patients QoL as well (Table 1).

### 3.3. Onychomycosis (OM)

This is very common disorder in Serbia significantly affecting patients QoL. Our laboratory- and hospital-based studies showed that OM is more common in toenails and is seen more frequently in males, while fingernail OM predominates in women. The mean prevalence of population- and hospital-based studies in Europe was 4.3% and 8.9%, respectively [13]. The exact data regarding prevalence in Serbia is missing. Using the European figures of 8.9%, we estimated a total number of 342,721 cases in our population older than 40 years (3,850,802 population size) corresponding to a prevalence of 4856/100,000. Both culture- and PCR-based surveillance of species causing OM showed the predominance of *Trichophyton rubrum* [14], and for fingernails *Candida* yeasts [15]. Infection with multiple fungi and non-dermatophyte molds is increasing in Serbia (NRL MM, http://nrl-eng.mikologija.org.rs).

### 3.4. Tinea Capitis (TC)

The disease TC or scalp ringworm is the most common dermatophyte infection of the scalp, affecting mainly children in Serbia. It may occur sporadically or epidemically. A total of 185 children were laboratory-diagnosed with TC in 2016, among them 110 in the age group 0–7 years, and 75 aged 7–14 (population size 1,016,579) which is a prevalence of 18.2/100,000 in the Serbian population <14 years. Children aged less than 7 years of either gender remain the most commonly affected but increasing numbers of cases have been observed in adults. Most of the affected adults are immunocompromised, and the real expected prevalence for TC is about 29.5/100,000 in the Serbian population <14 years (~300 cases). Laboratory-based surveillance showed most cases caused by *Microsporum canis* (41%), *T. mentagrophytes* (36%) and *M. audouinii* (23%) were in children [16]. According to the literature estimates [17], we expect the total number of fungal infections of the nails, skin, or hair to be 1,008,625 cases, a prevalence of 14,290/100,000 (14.3% of the population in Serbia).

### 3.5. Fungal Keratitis (FK)

The single tertiary care center-based survey of FK in the Clinical Center of Serbia, the Clinic for Ophthalmology, showed sporadic cases of proven FK limited to 10 to 12 cases per year, mainly caused by *Fusarium*, *Aspergillus*, and *Alternaria* [18]. However, using a recent estimate from Italy [6] and considering that approximately 41,000 of Serbian people wear contact-lenses, our estimation is a total number of ~70 cases per year, corresponding to a rate of 1/100,000.

### 3.6. Oral and Esophageal Candidiasis (OC, EC)

*Candida* mucosal infections are one of the most common fungal conditions in Serbia, especially in newborns, people living with HIV/AIDS, diabetes mellitus or cancer, as well as people who wear dentures or take antibiotics or corticosteroids, similar to published data [19,20,21].

Serbia has a low prevalence of HIV/AIDS; the first case was diagnosed in 1985. To date, a total of 3664 patients were diagnosed with HIV infection and 1901 developed AIDS. So far 1223 patients have died, leaving a total of 2441 living with HIV now, almost 90% on anti-retroviral treatment (ART) [19,20]. In the HIV cohort without ART (244 patients) and in the newly diagnosed cases (average ~130 per year) we may expect 332 and 42 cases of OC and EC, respectively, corresponding to an incidence of 4.7/100,000 and 0.6/100,000 respectively. In addition, the total of cancer patients in Serbia was estimated at 42,221 cases the GLOBOCAN 2012 database [22], among them we may expect at least 131 cases of EC (1.6/100,000 rate), according to the literature (0.32%) [23]. Almost 10% of the overall population are living with diabetes mellitus (~705,832 cases in Serbia) [24] and we estimated ~10% having OC among them, representing ~70,583 patients. Serbia has a large older population; almost 20% are older than 65 years (1,352,948 population size). In this group about 15% of the population wear dentures (202,942 cases), and our laboratory based investigation showed a prevalence of *Candida* stomatitis of 32.8% [25], which makes for 66,991 OC cases in denture wearers. Also, severe *Candida*-related prosthetic stomatitis (Newton classification—severity type III) was found in 18.3% cases and was mainly associated with mixed *Candida* spp. or NAC strains which can be difficult to treat [25]. The incidence data for OC following antibiotic or corticosteroid usage is difficult to estimate. Therefore, OC and EC were estimated in 208,489, and 173 cases, corresponding to 2954/100,000 and 2.45/100,000 annual incidence, respectively.

### 3.7. Recurrent Vulvovaginal Candidiasis (RVVC)

The exact number of women of fertile age with ≥4 episodes of *Candida* infection per year in Serbia is not known. However, a prevalence of RVVC between 6% and 9% has been reported in women aged 15 to 55 years [19] and this population size in Serbia is 1,804,043. Using a 7.5% prevalence, 135,303 cases RVVC cases are estimated, a prevalence of 3737/100,000 in all females as the denominator (Table 2).

Culture based laboratory surveillance of vulvovaginal *Candida* spp. prevalence showed an emergence of non-*albicans Candida* (NAC), which we identified in ~25% of Serbian isolates [26].

### 3.8. Respiratory Fungal Diseases

Chronic respiratory diseases are very common underlying conditions for SFD in Serbia. COPD is estimated in 6.5% of those over 40 years of age (population size 3,850,802), mainly smokers, so a total of 250,302 COPD cases. Among them, 16,742 were hospitalized in 2015 [27,28]. Asthma is present in 4% of students and in 5.6% of older people (population size 5,686,085) [27], an estimated total of 311,806 asthma cases. There were 7514 hospital additions for asthma in 2016 (IPHS, http://www.batut.org.rs). In addition, pulmonary sarcoidosis prevalence is relatively high in Serbia at 1120 cases corresponding to a prevalence of 16/100,000 [29]. The number of CF patients is 221, including 65 adults [29]. According to the literature chronic rhinosinusitis (CRS) affects about 10.9% of adults worldwide [30]. We estimated 658,499 cases of CRS among population size of 6,041,743.

As a consequence of these chronic respiratory diseases, fungal respiratory diseases may develop such as SAFS, CPA, fungal rhinosinusitis (FRS), and various fungal conditions in CF. ABPA is most common in adult asthmatic and adult CF patients. Using a figure of 2.5% [31], a cohort of 9026 adults with ABPA is estimated in Serbia, and in addition, 18% of adults (12 cases) and 11% of children with CF (16 cases) may have ABPA. Among adult asthmatics, poorly controlled and problematic asthma is thought to affect 10% or 31,181 cases, and fungal sensitization is more common in this population. Using Greek fungal sensitization data (32% of poorly controlled asthmatics) [32], we estimate that 10,393 asthmatics have SAFS in Serbia. There may be some duplication of patients between ABPA and SAFS as some ABPA patients have severe asthma and *A. fumigatus* sensitization is common among SAFS patients—estimated at 20% overlap. Among 1120 cases of pulmonary sarcoidosis we expect 67 cases of CPA, according to the literature [33].

As about 10% of adults with CRS may suffer from FRS (allergic and saprophytic (fungus ball of the sinuses)) [34], we estimated a prevalence of allergic FRS in 65,849 cases or 933/100,000 rate.

### 3.9. Life Threatening Fungal Diseases

Invasive candidiasis (IC), invasive aspergillosis (IA), and *Candida* or *Fusarium* BSI are responsible for >80% of life-threatening SFD in Serbia. As in other countries, these fungal infections occur mostly in ICU, renal support, abdominal surgery, hematology/oncology, transplant, neonatal settings, HIV, TB, and some respiratory conditions such as asthma, COPD, and CF.

### 3.10. Intensive Care Unit (ICU) Including Abdominal Surgery, and Renal Support

Based on the 1682 ICU beds, which often include patients following abdominal surgery, we estimated a total of 19,821 ICU patient admissions per year [35]. In our first multicenter laboratory based report of *Candida* BSI in a Serbian ICU, we found an incidence rate of 4 proven cases/1000 admissions (observed in 10,820 patient admissions), and accordingly we expect 80 proven *Candida* BSI in a Serbian ICU annually (rate 1.15/100,000) [3]. As about 30% of cases of candidaemia are documented in ICU in most other countries, the overall annual incidence would be about 3.8/100,000. This is a low estimate compared to other European population rates (3 to 10/100,000 cases) [36] or to the Italian ICU study which found the rate for yeasts BSI of 16.5/1000 admissions [37]. Extrapolating these estimates to Serbia we can expect 216 to 720 ICU candidemia cases, an average of 468 cases annually. Candidaemia underestimates invasive forms as blood cultures and are often (>50%) falsely negative.

Also, *Candida* peritonitis or intra-abdominal candidiasis occurs in our ICU patients, as well as in the patients under chronic ambulatory peritoneal dialysis (CAPD). We estimate 157 cases of *Candida* peritonitis/intra-abdominal candidiasis in ICU each year (2.24/100,000) using figures from France [37] and Hungary [7]. In cohort of 520 CAPD patients we presume an additional 30 intra-abdominal *Candida* cases (0.43/100,000) each year. In addition, in these settings we also anticipate invasive infections due to Mucorales and *Fusarium* at least in 3 cases, and *Pneumocystis* pneumonia in 45 cases yearly (Table 2).

### 3.11. Haematological Malignances (HM), Cancers, and Transplants

A total number of 2047 new HM cases were reported in 2015 (29.2/100,000) (IPHS, http://www.batut.org.rs/). Acute myeloid leukemia (AML), stem cell transplants (SCT), and solid organ transplants (SOT) represented 212, 151, and 59 cases, respectively [38]. The low number of SOT is due to the fact that the majority of transplantations were done abroad and local data is incomplete. Among the total number of 42,221 solid organ cancers (602.3/100,000), the prevalence of lung cancer is the highest in Europe and accounts for 7260 cases (103.6/100,000) [39]. These risk groups together are a total of 44,268 patients at risk for SFD but an increase is expected in 2019 due to the carcinogenic effect of depleted uranium [40], which will be maximal 20 years after the 1999 NATO air strikes in Serbia [41].

*Candida* or *Aspergillus* invasive infections are expected in HM and transplant patients, and account for about 50 and 41 cases yearly, respectively, based on estimates from Italian confirmed fungal cases [36]. The epidemiological survey on invasive infections due to primary plant mold *Fusarium* species in Europe showed the highest number of proven BSI among Serbian HRP, mainly children [42]. In addition, in these settings we expect invasive infections due to Mucorales and *Fusarium* in at least 20 cases, and *Pneumocystis* pneumonia in 2 cases yearly. The high number of lung cancers in Serbia probably translates into as many as 257 IPA cases annually, using data from China which found an incidence of 2.36% [43], because data from Europe is lacking.

A few cases of rare yeasts, molds, and mixed fungal infections are expected in our HRP, especially during neutropenia [44] (Table 2).

### 3.12. Human Immunodeficiency Viruses (HIV) and Tuberculosis (TB)

The number of HIV/AIDS cases not under regular follow-up and ART, and new HIV cases in Serbia are 244 and ~130, respectively. Among them, the most common life threatening fungal diseases are *Cryptococcal* meningitis [45], and *Pneumocystis* pneumonia, which we expect in 5 and 15 cases per year, respectively. We do not have good estimates for cryptococcosis in non-HIV patients. In 2015, Serbia reported 898 cases of pulmonary tuberculosis (PTB) to the WHO and it is assumed that 90% survived with only 6 cases in HIV patients in 2014. Using previously published means of estimation (a 12% of these with a cavity following PTB and 2% in those without a cavity) [46], the annual incidence of subsequent CPA is 36 cases and the 5-year period prevalence is 112 patients (Table 2).

### 3.13. Aspergillosis in Chronic Obstructive Pulmonary Disease (COPD) and Sarcoidosis 

Using a 5-year prevalence of CPA after PTB of 112 cases, and assuming that this underlying disease is responsible for only 20% of all CPA cases in Serbia (as in the UK) [47], we expect a total prevalence of 448 CPA cases in Serbia, or 6.35/100,000 rate. Besides 112 PTB cases, this includes 67 cases in sarcoidosis, and 345 COPD cases. Using incidence data for IA based on positive culture reported from Madrid (1.3%) [48], an anticipated 221 COPD patients are likely to develop IPA (probable or possible) annually (Table 2). Individual cases of lung or sinus fungus ball (simple aspergilloma), mucormycosis, *Fusarium*, *Aspergillus terreus*, *Stachybotris chartarum*, or mixed mold infections are also expected in these settings [44,49].

### 3.14. Documented Fungal Outbreaks in Serbia

Several high-profile fungal outbreaks have been documented in Serbia as an increasingly important health problem, and occur both in the community and health care settings. The complete data for these outbreaks are missing, as well as the publications, because they are usually under-investigated and under-reported. Interestingly, most of these outbreaks occur among children. The first report of a *Rhinosporidium seberii* outbreak in Europe comes from Serbia in 1993 and was associated with water from Silver Lake and the environmental conditions. More than 20 children were affected over two years and the number of patients in this epidemic exceeded the total number of autochthonous cases of rhinosporidiosis ever recorded in Europe till then [50]. A *C. albicans* nosocomial outbreak occurred in a Maternity hospital in Belgrade during 2009 and in 11 neonates *Candida* BSI were diagnosed and complicated with osteomyelitis [51]. In 2012 another healthcare outbreaks occurred in pediatric oncology settings in Belgrade and in Novi Sad, and more than 10 children were affected with *Fusarium* spp. which seems to be an emerging and important pathogen in children associated primarily with the north part of Serbia (province Vojvodina) [42].

In addition, a *C. tropicalis* outbreak was documented in two adults with HM in 2013, and several *Aspergillus* associated outbreaks at hematology settings have been clinically documented during building reconstruction in Serbia hospitals [52], and aspergillosis in a flock of turkey poults [53].

### 3.15. Rare, Emerging, or Antifungal Resistant Species in Serbia

Documented deep tissue or BSI observed with rare/emerging species are: *Scedosporium apiospermium* (*Pseudallesheria boydii*), *Fusarium proliferatum*, *F. oxysporum*, *F. verticillides*, *A. terreus*, *Stachybotris chartarum*, *Geotrichum candidum*, *Saccharomyces cerevisiae*, *Cryptococcus*, *Pneumocystis* and *Mucorales* [42,44,54]. A presence of *Schizophyllum commune* in a skull base was described in an immunocompetent patient with CRS [55]. A few mycetoma and fungus ball cases have been observed [49]. Antifungal and azole cross-resistance species observed in Serbia are associated with a high mortality rate, which we estimated at least in 409 deaths annually (rate 5.79/100,000) [3].

*Candida auris*, chromoblastomycosis, sporotrichosis and histoplasmosis have not been documented yet in Serbia.

## 4. Discussion and Conclusions

For the first time in the literature we present an epidemiological estimation of SFD in Serbia. Historically, the first population and laboratory-based survey for dermatomycoses was done in Belgrade in 1930 [56], and followed by a critical survey of mycological data regarding dermatomycoses up to 1957 [57]. Both papers were refering to Yugoslavia, because Serbia was part of this country. Recently, the first multicenter report of a national prospective observational survey for *Candida* BSI in Serbia was published as the result of SSMM and NRL MM activities [3].

Although this study represents a rough estimation of the burden of SFD in Serbia, it can be assumed that at least 1 million people (16.5% of the total population) might be affected: 14.3% with chronic and allergic fungal diseases, which affects QoL, and 2.2% with fungal infections that threaten life. In addition, a mortality rate of 5.8/100,000 cases annually is expected due to the results of our first estimation on *Candida* BSI in ICU [3]. But the real mortality is extremely difficult to determine due to the lack of the obligatory reporting of fungal-related deaths in Serbia.

In this study a low number of TC, FK, and cryptococcal meningitis cases were estimated. Beside the fact that TC had been abundantly recognized between the First and Second World War in Serbia, nowadays population based surveillance is absent. The epidemiology of TC in children, adolescents and adults has changed over the last few decades, indicating the needs for nationwide surveillance in Serbia. Therefore, the low number of hospital based culture-proven TC cases and estimation seem to be highly underestimated in this study. The low number of culture-proven FK probably represents a real under-estimation of FK incidence in Serbia, which can most likely be explained by difficulties in sampling to get good specimens. However, progress has been made by educating contact-lens wearers and by contact-lens liquid screening for fungal contamination. The annual incidence of opportunistic fungal infections in HIV-positive patients is low, having a similar or even a reduced incidence compared with other EU countries or previous report [45].

However, a high number of IPA, and CPA in COPD, lung cancer, and patients with sarcoidosis are estimated. These estimates show that respiratory underlying conditions are common in Serbia and are the main predisposing factors for SFD, especially for CPA and IPA (Table 2). Cancer incidence in Serbia is much higher compared to the EU region, especially for lung cancer, which showed an alarming incidence of 103/100,000 cases, and further increase is expected. The prevalence of asthma and COPD are estimated to be 4.5% and 3.5% among whole population (Table 1), which may explain the high presence of ABPA and SAFS in the Serbian population.

Documented invasive fungal infection outbreaks in Serbia stress the importance of education and prevention as a key aspect of SFD [58,59]. Serbia has agriculture rich regions in its Northern part, and we presume that this is associated with a high number of *Fusarium* cases or outbreaks related to exposures in this region. Emerging features of fungal infection outbreaks in Serbia include new at risk populations such as children, and unexpected pathogens in geographical areas where they have not previously been recognized; e.g., *Rhinosporidium seeberi*. This outbreak was associated with lake water exposure. Therefore, the many stagnant waters in Serbia may contribute to specific environmental conditions for fungal growth, possibly resulting in new outbreaks. Although *C. auris* has not been detected yet in Serbia, there is need for the establishing fungal surveillance routinely, adherence to health-care infection control practices, and public awareness, taking into account previous cases of neonatal *C*. *albicans* and *C. tropicalis* outbreaks in Serbia.

Early diagnosis based on fungal early laboratory biomarkers weekly screening in HM patients (galactomannan and mannan antigens) was established as a key Serbian NRL MM activity in 2008. As the result of ten years of activity, we formed a registry that includes 1581 adults and 625 pediatric HRP using clinical- and laboratory data [52]. According to preliminary analyses we observed galactomannan positivity in several patients with *Fusarium* BSI, as well as in several patients with mold mixed infections. In these cases poor responses to voriconazole treatment and cytology- and culture based supplementary diagnosis has led to diagnosis and improved outcomes [44].

Standard antifungals are available in Serbia now including liposomal amphotericin B, ketoconazole, miconazole, fluconazole, itraconazole, voriconazole, posaconazole, and echinocandins (caspofungin, anidulafungin, micafungin), while flucytosine and isavuconazole are lacking in Serbia (www.mikologija.org.rs; www.gaffi.org/who/our-ambassadors/serbia/).

This study has some limitations mostly due to the lack of official data. Additionally, the main drawback is that many of the results are not directly measured prospectively but are products of calculations and formulas. Because the estimates represent approximations, it is likely that true numbers will differ. However, these estimates will be useful to prioritize both research efforts and healthcare expenditures.

In conclusion, our data have shown that SFD are very common in Serbia; however, they are rather underestimated because of very few nationwide epidemiological studies. Based on local reports and prevalence estimation, we estimated a low number of TC, FK, and cryptococcal meningitis cases, and a high number of IPA and CPA in COPD/lung cancer, and in sarcoidosis, respectively. In addition, Serbian fungal disease epidemiology has certain features, distinct from that of our region and the World and a precise epidemiological overview of SFD in Serbia is only possible by putting into practice a prospective multi-annual study. Invasive fungal infections or SFD are still unsuspected, undiagnosed, and untreated, even though most of the deaths from them could be avoided, therefore an obligatory registration by health authorities is necessary.

## Figures and Tables

**Table 1:** Demographic characteristic and size of population at risk of serious fungal diseases in Serbia.

Population Structure ^#^	Number (n)	Percentage (%) of Total Population in Serbia
Total population	7,058,322	100
Adults (>15 y)	6,041,743	85.60
Adults (15–65 y)	4,688,795	66.43
Adults (>40 y)	**3,850,802**	54.56
Adults (>65 y)	**1,352,948**	19.17
Children (˂14 y)	**1,016,579**	14.40
Children (7–14 y)	544,632	7.72
Women (all ages)	3,620,692	51.30
Women (15–55 y)	**1,804,043**	25.59
**Size of population at risk for selected serious fungal diseases (SFD) in Serbia**		
Asthma	311,806	4.42
COPD	250,302	3.55
Cancer—lung *	7260	1.02
Sarcoidosis	1120	0.16
CF in adult	65	0.00092
AML	212	0.003
Allogeneic SCT	50	0.00071
SOT	59	0.00084
HIV not under ART *	244	0.00346
Tuberculosis pulmonary *	898	0.01272

^#^ Source: РЗС. Становништво [11]; The numbers of the populations that are most predisposed to SFD are bolded. Abbreviations: AML—Acute myeloid leukemia; ART—anti retroviral therapy; CF—cystic fibrosis; COPD—chronic obstructive pulmonary disease; SCT—stem cell transplant; SOT—solid organ transplant; TB—tuberculosis, HIV—human immunodeficiency virus, y—years; * Total cases in 2016: Cancer 42,221 (0.167%); HIV 2441 (0.035%); TB 962 (0.0136%); Intensive care unit admissions 19,821 (3.99%); Renal support 520 (0.007%); chronic rhinosinusitis 658,499 (9.33%); diabetes mellitus 705,832 (0.01%); denture wears 202,942 (0.003%).

**Table 2:** Yearly incidence estimated for serious fungal diseases (SFD) in Serbia: population in 2016.

Disease	Number of Infections per Underlying Disorder per Year					Rate/100,000	Total Burden
	None	HIV/AIDS	Respiratory	Cancer/Chemotherapy	ICU		
Oesophageal candidiasis-EC	-	42	-	131	-	2.4	173
Candidaemia	-	-	-	50	468	7.3	518
*Candida* peritonitis	-	-	-	-	187 ^#^	2.7	187
Recurrent vulvovaginal candidiasis-RVVC (4×/year+)	135,303	-	-	-	-	3737 ^##^	135,303
Allergic bronchopulmonary aspergillosis-ABRA	-	-	9094	-	-	129	9094
Severe asthma with fungal sensitization-SAFS	-	-	10,393	-	-	147	10,393
Chronic pulmonary aspergillosis-CPA ^a^	-	-	448	-	-	6.4	448
Invasive aspergillosis-IA ^b^	-	-	478	41	100	8.8	619
Mucormycosis, *Fusarium*	-	-	-	20	3	0.33	23
Cryptococcal meningitis	-	5	-	-	-	0.07	5
*Pneumocystis* pneumonia	-	15	-	2	45	0.88	62
**Total burden estimated**	**135,303**	**61**	**20,413**	**245**	**821**	**2221**	**156,825**

**^#^** include cases from abdominal surgery and peritoneal dialysis; ^a^ include cases from pulmonary tuberculosis—PTB, chronic obstructive pulmonary disease—COPD, and sarcoidosis; ^b^ includes cases from lung cancer, COPD, hematological malignancy—HM, and intensive care units—ICU; ^##^ All females as the denominator; Additional SFD estimated in Serbia: fungal keratitis ~70; tinea capitis ~300; onychomycosis 342,721; oral candidiasis 208,489; fungal rhinosinusitis 65,849.
